# Concentrated Growth Factors and Bone Grafting in Maxillary Sinus Lift: A Systematic Review

**DOI:** 10.1155/ijod/6681492

**Published:** 2025-12-03

**Authors:** Antonio Mancini, Francesco Inchingolo, Angelo Michele Inchingolo, Vincenzo Carpentiere, Gaetano Del Vecchio, Andrea Palermo, Alessio Danilo Inchingolo, Gianna Dipalma

**Affiliations:** ^1^Department of Interdisciplinary Medicine, University of Bari “Aldo Moro”, Bari 70124, Italy; ^2^University of Salento, Lecce 73100, Italy

**Keywords:** allograft, growth factor (GF), sinus lift (SL), surgery

## Abstract

**Aim:**

This review evaluates the latest studies on the effectiveness of growth factor concentrates and bone grafts (BGs) in maxillary sinus lift (MSL) procedures, emphasizing their role in bone regeneration and healing. The aim is to determine their impact on enhancing clinical outcomes by improving bone quality and accelerating the healing process.

**Materials and Methods:**

A comprehensive literature search was conducted using the Scopus, Web of Science, and PubMed databases. The following keywords were employed: “PRP,” “L-PRF,” “CGF,” “oral surgery,” and “sinus lift,” combined with the Boolean operators “AND” and “OR.” A total of 727 studies were initially identified, of which 18 met the inclusion criteria for this review.

**Results:**

The included studies demonstrate that the use of growth factors in conjunction with alloplastic and xenogeneic BGs significantly promotes early vascularization and has potent proangiogenic effects in vivo. Furthermore, these combinations reduce postoperative inflammation and discomfort, accelerating the healing process and enhancing tissue regeneration.

**Conclusions:**

Although further research is necessary, current evidence suggests that autologous platelet concentrates hold promise for improving clinical outcomes in MSL procedures. They may enhance bone height and thickness, improve vascularization, and expedite postoperative healing, offering a valuable adjunct to traditional bone grafting techniques in oral surgery.

**Summary**

What is known:

• Growth factor concentrates and bone grafts (BGs) are commonly used in maxillary sinus lift (MSL) procedures.
• There is increasing interest in their role in bone regeneration and healing.

What this study adds:

• This review evaluates the effectiveness of growth factor concentrates and BGs in enhancing clinical outcomes in MSL procedures.
• Findings suggest that autologous platelet concentrates may accelerate healing and improve bone quality.

## 1. Introduction

Implant-prosthetic rehabilitation is a cornerstone of modern dentistry, focusing on the restoration of masticatory, esthetic, and phonetic functions compromised by partial or total tooth loss [1, 2]. This therapeutic approach involves the use of dental implants, which serve as artificial roots to support fixed or removable prostheses, enabling patients to regain effective chewing, confident smiling, and smooth speech [3, 4]. Implant-prosthetic solutions are versatile, with applications ranging from fixed total dentures to removable superstructures, all aimed at providing stability and comfort to the patient [5–10]. Additionally, implants play a crucial role in preventing bone resorption and maintaining jawbone health, particularly in cases of tooth loss due to trauma or decay [11–18]. However, in the posterior regions of the maxilla, severe bone atrophy often occurs due to sinus pneumatization or previous osteolytic conditions [19–22].

**Figure 1:**
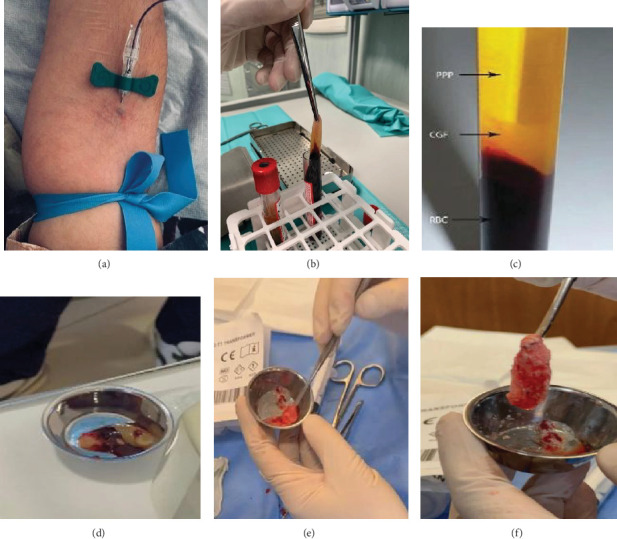
Preparation and mixing with allograft. (a) Venous sampling; (b) centrifuged venous blood; (c) detail of centrifuged blood; (d) PRF and CGF; (e) PRF and CGF mixed with allograft; and (f) PRF and CGF mixed with allograft. The figure is an original figure created by the authors of the manuscript.

In the context of sinus augmentation, two primary surgical approaches are commonly employed: the lateral approach and the crestal approach [23–43]. According to recent clinical guidelines, the choice between lateral and crestal approaches should not rely solely on the residual crestal bone height, but also consider qualitative aspects of the residual bone, such as cortical thickness and bone density. Stacchi et al. [44] proposed a decision tree that emphasizes the role of residual bone quality in determining the most appropriate surgical technique. The lateral approach, or “lateral window technique,” involves creating a window in the lateral wall of the maxillary sinus to allow for significant bone augmentation. This method is particularly indicated when there is minimal residual crestal bone (2–3 mm) [45–51]. Although effective, the lateral approach is more invasive and carries a higher risk of complications, such as sinus membrane perforation [52–60]. On the other hand, the crestal approach, also known as “transcrestal sinus lift (SL),” is less invasive and is preferred when only a small amount of bone augmentation is needed (4–5 mm of residual crestal bone). This technique involves creating a small hole through the alveolar ridge and gently lifting the sinus membrane [61–65]. Bone graft (BG) material is then introduced through the hole, and in some cases, the dental implant can be placed simultaneously [66–70]. The crestal approach is associated with faster recovery times and a lower risk of complications compared to the lateral approach [71, 72]. The choice between these two techniques should be based on a structured clinical decision-making process that takes into account not only the residual bone height (RBH) but also the bone quality, anatomical factors, and overall treatment plan [44, 73]. Current literature suggests following standardized protocols and decision trees, such as those proposed by Stacchi et al., to improve outcomes and minimize complications [44, 74–76]. Despite the advantages of SL techniques, several research limitations persist [77]. Clinical outcomes can vary significantly due to individual differences in bone quality, healing capacity, and graft response [78–81]. Complications such as sinus membrane perforation and infection remain as primary concern that can impact implant success and longevity. In the realm of bone regeneration, there is growing interest in the use of autologous platelet concentrates, such as concentrated growth factors (CGFs), platelet-rich fibrin (PRF), and platelet-rich plasma (PRP) [82–89]. These concentrates, rich in growth factors, have shown promise in promoting the regeneration of both soft and hard tissues, thereby enhancing the healing process and supporting bone regeneration. The unique biocompatibility and regenerative potential of PRP, PRF, and CGF make them valuable adjuncts in sinus augmentation procedures (Figure 1). In the context of maxillary sinus augmentation, CGFs have shown promise in improving clinical outcomes by accelerating the healing process and enhancing the quality of newly formed bone [90, 91]. When used in combination with BG materials, whether autologous, allogeneic, xenogeneic, or synthetic, CGFs promote early vascularization, which is critical for the survival and integration of the graft. The growth factors present in CGFs, such as transforming growth factor-beta (TGF-*β*), vascular endothelial growth factor (VEGF), and platelet-derived growth factor (PDGF), play pivotal roles in modulating inflammation, stimulating angiogenesis, and enhancing the recruitment of osteoprogenitor cells to the graft site. Furthermore, the use of CGFs in SL procedures has been associated with a reduction in postoperative complications. The anti-inflammatory properties of these growth factors help minimize the risk of infection and reduce patient discomfort during the recovery period. Additionally, CGFs can potentially decrease the incidence of sinus membrane perforation by promoting faster and more effective membrane healing [63, 64, 92–95]. The integration of CGFs into sinus augmentation protocols not only optimizes the regenerative environment but also allows for the use of less invasive techniques, potentially reducing the need for extensive grafting procedures.

Therefore, continued research is essential to address these challenges, minimize complications, and improve long-term outcomes, ultimately leading to safer and more effective treatment modalities. The present systematic review aims to provide a comprehensive overview of the latest research on the use of CGFs in SL procedures. Specifically, it will analyze studies that investigate the effects of CGFs on bone regeneration, healing, and clinical outcomes. Additionally, the review will evaluate the efficacy of using CGFs in combination with various graft materials, including allografts, xenografts, and synthetic grafts, for sinus augmentation. The goal is to determine how these combinations influence early vascularization, reduce inflammation, enhance postoperative healing, and contribute to overall regenerative success.

## 2. Materials and Methods

### 2.1. Protocol and Registration

The Preferred Reporting Items for Systematic Reviews and Meta-analysis (PRISMA) guidelines were followed in the conduct of this research [96]. It has been registered under the provisional number 551488 on The International Prospective Register of Systematic Reviews (PROSPERO).

### 2.2. Research Processing

The Boolean operator “AND” was utilized in conjunction with the search phrases “(SL), oral surgery, GF, and allograft” in the databases PubMed, Web of Science, and Scopus to select papers for review.

The search was limited to only articles published in English within the following time interval: 2014–2024 (Table 1).

### 2.3. Eligibility Criteria

The reviewers, who worked in pairs, chose papers that met the following inclusion criteria: (1) research exclusively on human subjects, (2) clinical studies or case, and (3) articles focused on the analysis and evaluation of sinus augmentation techniques, with a focus on the use of autologous growth factor concentrates to improve bone regeneration and clinical outcomes. Exclusion criteria were (1) animal studies; (2) in vitro studies; (3) narrative reviews, systematic reviews, and meta-analyses; and (4) studies away from the topic of inclusion.

The review was conducted using PICO criteria as follows:

– Population: adults, both male and female, requiring SL.
– Intervention: biomaterials and growth factors used in the SL technique.
– Comparison: SL technique with and without growth factors.
– Outcome: effectiveness in SL.

### 2.4. Data Processing

Excluding any publications that did not align with the issues reviewed was made feasible by the screening process, which involved examining the abstracts and titles of the papers selected during the identification step. After that, the full texts of the articles judged to fit the preset inclusion criteria were examined. Reviewers' disagreements on article selection were discussed and resolved.

### 2.5. Quality Assessment

The quality of the included papers was assessed by two reviewers, Vincenzo Carpentiere and Gaetano Del Vecchio, using the ROBINS-I tool. In order to assess potential bias in the results of nonrandomized studies evaluating the effects of two or more medicines on health, ROBINS-I was developed. Every one of the seven assessed points received a biased degree. Until a consensus was formed, disagreements were discussed with Francesco Inchingolo, the third reviewer. Reviewers were trained to evaluate potential bias in seven domains: confounding, participant selection, intervention classification, deviations from intended interventions, missing data, outcome measurement, and choice of reported results. They also followed guidelines when using the ROBINS-I tool in order to increase the assessments' impartiality and consistency; disagreements or disputes among assessors were resolved through discussion and consensus. A third reviewer made the ultimate judgment in cases when a consensus could not be achieved. ROBINS-E was used for bias assessment, which allowed for a thorough evaluation of potential biases in the nonrandomized trials included in this analysis. It made the benefits and drawbacks of the evidence base clear, which improved the assessment of the overall quality and reliability of the results. By taking the possibility of bias into consideration, the authors of this review were able to draw more well-informed interpretations and conclusions from the available data.

## 3. Results

**Figure 2:**
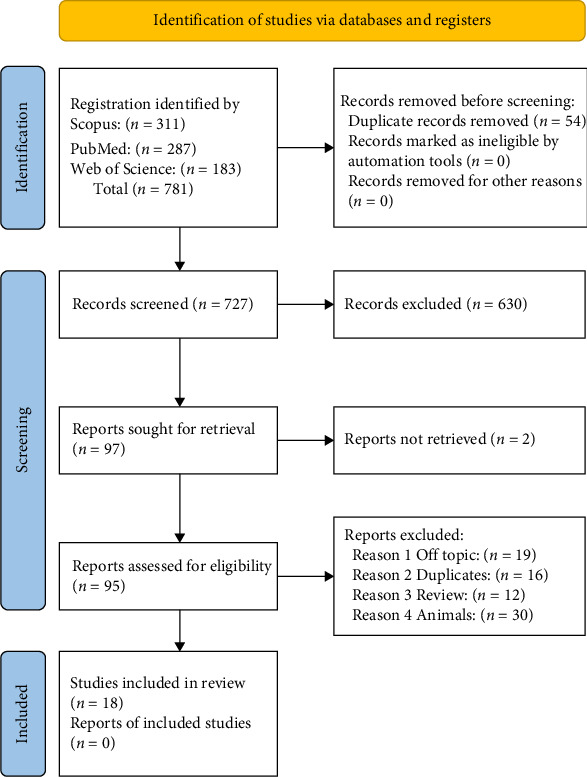
PRISMA flowchart [96].

Using keyword searches, the Web of Science (183), Scopus (311), and PubMed (287) databases yielded a total of 781 papers. After removing duplicates (a total of 54 articles), 727 articles remained. 630 papers from these 727 studies were excluded due to their noncompliance with the preestablished inclusion criteria. Following the selection of 29 papers for this study, the screening procedure was completed (Figure 2). Each study's findings were presented in Table 2.

### 3.1. Bone Formation Outcomes

• Adalı et al. [98] found increased new bone formation when CGF was added to allografts.
• Dominiak et al. [105] reported effective bone formation with PRF over 3 years.
• Taschieri et al. [113] found no significant difference in viable bone between PRP and control.

### 3.2. Implant Survival

• Khouly et al. [103] noted that high implant survival correlated with PRGF use.
• Chen et al. [104] reported 100% success with osteotome sinus floor elevation (OSFE) and CGF.

### 3.3. Soft Tissue Healing

• Gurler and Delilbasi [99] showed minor improvements in healing with leukocyte-PRF (L-PRF).
• Sher Ling [100] reported enhanced healing using liquid CGF as a membrane.

### 3.4. Complications and Morbidity

• Ntontoulos and Dabarakis [108] observed reduced postoperative morbidity using Alb-CGF.
• Rațiu et al. [114] described initial complications followed by bone regeneration with PRGF.

### 3.5. Quality Assessment and Risk of Bias of Included Articles

**Figure 3:**
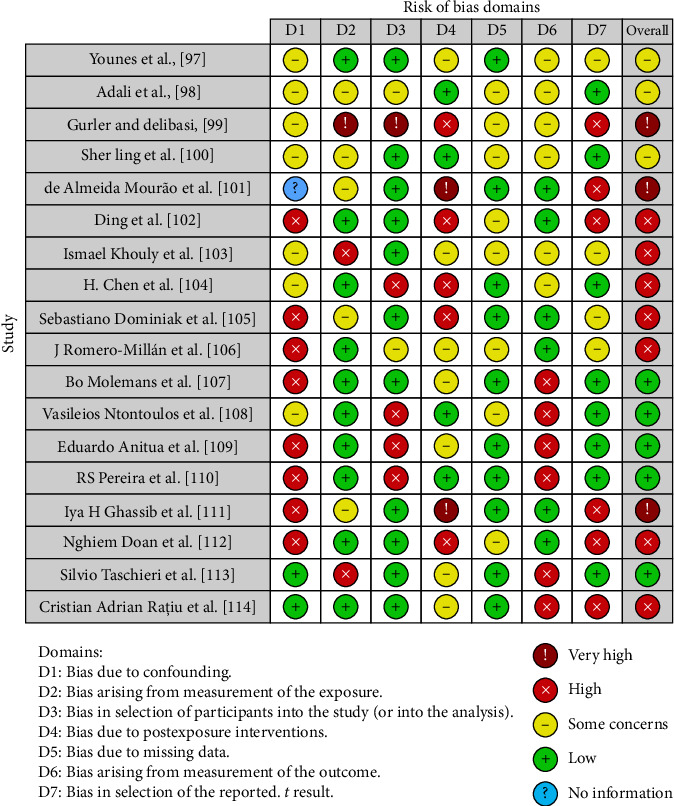
Bias evaluation with the Robins tool.

Figure 3 indicates the risk of bias in the selected studies. Confounding bias is a major source of bias in most research. One parameter that has little chance of bias is measurement bias. A low risk of bias in many research can be attributed to participant selection bias. The significant variability makes it impossible to assess bias resulting from postexposure. Many studies have low bias rates because of missing data. There is little bias resulting from outcome measurement. In most research, there is a significant bias in the selection of the published results. According to the findings, there is a low risk of bias in five studies, a very high risk of bias in three, and a high risk of bias in eight.

## 4. Discussion

Sinus floor elevation is a surgical procedure designed to increase bone height in the posterior region of the upper jaw to facilitate the placement of dental implants [113]. This technique is particularly useful in patients with significant bone atrophy. In recent years, the use of CGF, such as PRP, PRF, and L-PRF, combined with BGs has shown promising clinical results [108, 115].

A study by Younes et al. [97] investigated the use of bovine deproteinized bone (deproteinized bovine bone mineral [DBBM]) as a filler material for SL. This prospective study found that despite a significant decrease in graft volume within the first 3 months, the graft volume stabilized thereafter, maintaining nearly 80% of the original volume at 2 years [97, 116]. Patient-reported outcome measurements indicated that postoperative discomfort, mainly pain, swelling, and hematoma, was common initially but decreased significantly over time. All placed implants showed good integration and favorable clinical outcomes during the 2-year follow-up, supporting the use of DBBM as a reliable filler material for these procedures [104, 117, 118]. The use of CGF has been examined in some studies. A Ling's case report describes the effectiveness of CGF in promoting healing of large bone defects when combined with BG [100, 119–121]. This protocol has shown promising results in soft tissue closure and bone regeneration, highlighting the ability of CGF to protect the sinus membrane and promote cell proliferation. In a prospective study by Molemans et al., L-PRF's effectiveness as the only graft material for sinus floor elevation and concurrent implant implantation was assessed [104, 107, 122]. The results showed an average vertical bone gain of 3.4 ± 1.2 mm for transalveolar elevation and 5.4 ± 1.5 mm for lateral elevation, with all implants clinically integrated after 6 months. This demonstrates the potential of L-PRF in promoting bone formation and stabilizing implants. Mourao et al. explored the use of nanostructured carbonated hydroxyapatite (cHA) microspheres combined with blood-derived growth factors (BDGFs) for sinus floor elevation [123, 124]. The results indicated that although BDGF improved surgical maneuverability, it did not significantly affect bone repair after 6 months. Hydroxyapatite microspheres showed promising results for bone formation, suggesting the need for further investigation into the impact of BDGFs [101, 106, 125]. Adali et al. [98] conducted a study comparing CGF mixed with allografts versus allograft alone. The results showed that CGF accelerated bone formation and maintained stability of augmentation, although histomorphometry differences were not significant [109, 110]. This study underscores the potential of CGF in bone regeneration, although larger-scale research is needed to confirm the results. Another novel approach was reported by Ding et al. [102], who used BMP2-loaded calcium phosphate grafts for. This study showed an average bone gain of 6.8 mm with stable implant function and minimal complications over 4–5 years. The results suggest that BMP2-loaded calcium phosphate is suitable for MSLS, especially in patients with minimal bone height [119, 121]. Khouly et al. highlighted PRGF use in combination with grafting materials to improve bone regeneration and facilitate implant placement [103]. The study showed that PRGF supplementation improved implant survival with increased RBH, suggesting a potential clinical benefit [111, 112, 126, 127]. Taschieri et al. [113] investigated SL via lateral approach, comparing histologic and histomorphometric outcomes using pure PRP mixed with DBBM against DBBM alone. In this split-mouth design study involving six patients, no significant differences were observed in the percentage of vital bone between the test group (30.70%) and the control group (22.72%). Both methods supported implant success. Further studies with larger sample sizes were deemed necessary to confirm these results and explore the relationship between native bone height and vital bone formation. R. S. Pereira et al. compared beta-tricalcium phosphate (*β*-TCP) (chronOS) with autogenous BG in SL surgery. Three groups were evaluated: *β*-TCP alone, *β*-TCP mixed with autogenous bone (1:1), and autogenous bone alone. Histomorphometric and immunohistochemical analyses were conducted on biopsies taken after 6 months. Bone formation percentages in the *β*-TCP group were similar across regions, while the *β*-TCP + autogenous bone group showed lower values. The autogenous bone group had varied results, with high cellular activity and immature bone in the *β*-TCP + autogenous bone group, unlike mature bone in the *β*-TCP and autogenous bone groups. *β*-TCP demonstrated comparable behavior to autogenous BGs, indicating its efficacy as a replacement for bone [110]. I.H. Ghassib et al. explored the process of transalveolar sinus floor elevation (TSFE), which increases the maxillary sinus's bone height. The study demonstrated the effectiveness and stability of this technique, particularly when combined with short implants and growth factor-rich plasma. Patients undergoing this procedure showed stable bone gain over time. The procedure involved local anesthesia, incision to expose the site, creation of a lateral window in the maxillary sinus, and sinus floor elevation. Dental implants were then placed, followed by wound closure and postoperative care. An 8-month follow-up showed significant bone formation around the implant apex, with vertical gain. Combining a collagen membrane with human PDGF-BB (rh-PDGF-BB) yielded positive results, though further studies were needed to validate these results and assess long-term efficacy [111]. N. Doan et al. analyze TSFE as a technique for increasing bone height in the maxillary sinus, highlighting its effectiveness and stability, especially in combination with short implants and growth factor-rich plasma. An 8-month follow-up indicated significant bone formation around the implant apex, with notable vertical gain. Combining a collagen membrane with rh-PDGF-BB showed promising results, warranting further studies to confirm long-term efficacy and clinical applicability on larger scales [112]. J.R. Millan et al. examined dental implant placement in the posterior maxilla, comparing implants in native bone to those after SL. The retrospective study followed 163 patients for at least 5 years, recording bone loss, vertical bone gain, and implant success and survival rates. No significant differences in bone loss were found between groups, though vertical bone gain was greater in the delayed implant group than in the simultaneous implant group. The SL technique showed variable results but generally stabilized after the first year. Implant survival rates were similar across groups [106]. V. Ntontoulos and N. Dabarakis examined sinus floor elevation using the Piezo-albumin-concentrated growth factor (Alb-CGF) protocol, a procedure with minimum invasiveness for dental implant insertion with RBH less than 6 mm. This method avoids open lateral approaches, reducing morbidity and postoperative complications like Schneiderian membrane perforation and sinusitis. Five healthy patients were treated using L-PRF (CGF) combined with denatured albumin. Pre and postoperative CBCT scans showed a significant increase in bone height and bone density, indicating effective bone regeneration with no adverse events. The study concluded that the Piezo-Alb-CGF protocol is safe and effective for sinus floor elevation in patients with low RBH, improving implant stability and reducing patient discomfort [108].

Gurler et al. [99] examined the use of L-PRF in soft tissue healing after direct. Although the differences from the control group were not significant, L-PRF showed higher healing index scores, indicating potential benefits in wound healing and immune regulation. Raţiu et al. [114] presented a case of sinus membrane elevation using only PRGF and fibrin clot. Despite initial complications, such as a vestibular abscess, observation at 8 months showed significant bone regeneration, allowing reinsertion of implants and confirming the efficacy of PRGF. Dominiak et al. [105] compared various grafting materials, including DBBM, *β*-TCP, and autologous PRF, for implant rehabilitation in edentulous jaws. The results indicated the potential of PRF as the only graft material, although long-term efficacy remains a subject of ongoing research.

## 5. Conclusions

The use of autologous growth factors like PRP, PRF, and CGF with BGs enhances bone regeneration and soft tissue healing by improving early vascularization, reducing inflammation and pain, and accelerating tissue regeneration. These factors can increase bone height and thickness and enhance dental implant stability and integration. Their combination with alloplastic and xenogeneic materials is particularly effective, though outcomes vary based on individual differences in bone quality and healing. Some studies show no significant improvements, highlighting the need for more research.

## Figures and Tables

**Table 1:** Database search indicators.

Item screening strategy	Database: Scopus, Web of Science, and PubMed
	Keywords: “PRP,” “PRF,” “L-PRF,” “CGF,” “OS,” and “SL”
	Boolean variable: “AND” and “OR”
	Period: 2014–2024
	English language

**Table 2:** Item results.

Authors [reference number]	Type of the study	Aim of the study	Materials	Results
Younes et al. [97]	Prospective study with 24 months of follow-up [97]	Assess volumetric changes and clinical outcomes following sinus floor elevation using deproteinized bovine bone mineral (DBBM).	Sinus augmentation from the side wall in 22 patients using DBBM as the only filling material.	All implants integrated successfully and demonstrated healthy clinical conditions.
Adalı et al. [98]	Randomized controlled clinical trial [98]	Evaluate whether Sinus augmentation procedures using CGF mixed with allografts are better than using allografts alone.	10 patients with residual bone height between 1 and 3 mm in the maxillary posterior region for sinus augmentation from the lateral wall.	Use of CGF with allografts can help regenerate new bone and replace graft with new bone.
Gurler and Delilbasi [99]	Randomized controlled clinical trial [99]	To evaluate the effects of leukocyte-rich platelet fibrin (L-PRF) after sinus direct lifting procedure.	Twenty-four patients were divided into two groups. Allogenic bone graft and L-PRF combination were utilized as the graft materials in the first group. The lateral window was closed with L-PRF membrane. In the control group, the lateral window was sealed with resorbable collagen membrane, and allogenic bone was the only material used for grafting.	The use of L-PRF and allogeneic bone graft in combination with L-PRF membrane did not show statistically significant differences compared to the control group, although a slight trend toward improved postoperative complications (e.g., reduced pain and swelling) was observed.
Sher Ling et al. [100]	Case report	To evaluate whether healing of large bone defects improves by using liquid CGF with bone graft and solid CGF as soft tissue membrane.	Clinical case of SL with CGF and allograft. Consolidated bone graft. Implant was placed in a mature area, restored with an individual crown.	Positive postoperative result confirmed by blood-derived growth factors (BDGFs). CGF, rich in GF and stem cells, promotes healing in large bone defects, protecting the graft and improving its survival and tissue regeneration.
de Almeida Mourão et al. [101]	Randomized, controlled, clinical trial	Study evaluates the efficacy of BDGF with calcium phosphate in regenerating the maxillary sinus floor.	Clinical study of 10 patients: comparison between carbonated hydroxyapatite (cHA) and BDGF in regenerating sinus floor, evaluated after 180 days.	BDGF improves the surgical process but does not increase bone formation in sinus floor elevation with cHA.
Ding et al. [102]	Clinical Trial	Assessing the reduction of graft resorption in MSLS cases.	Study of 20 patients: MSLS with calcium phosphate/bone morphogenetic protein (BMP) 2 graft.	Positive results, implant stability, and good dental function after 4–5 years. BMP2 in calcium phosphate is promising for MSLS, suitable for patients with low bone height, preferring 1- or 2-stage techniques.
Ismael Khouly et al. [103]	Clinical trial	To determine the factors affecting implant survival after sinus augmentation with PRGF and grafting.	Study of 67 patients with 217 implants in 100 augmented sinuses.	Implant survival related to residual bone height, implant length and immediate loading. PRGF in sinus grafting is promising for atrophic posterior maxilla rehabilitation but requires confirmation by randomized clinical trials.
H. Chen et al. [104]	Retrospective study	The maxillary region's residual bone height (RBH) before surgery of 4–6 mm is compared to simultaneous implant implantation (SIP) with or without bone graft and osteotome sinus floor elevation (OSFE) with CGF in this study.	44 patients, OSFE with CGF and SIP of 60 implants. Similar lift height, bone resorption, and marginal bone loss were not significant, but greater pain with graft.	Implant success rate was 100% using OSFE with CGF, regardless of the use of bone graft material. This suggests the technique is safe and reliable.
Sebastiano Dominiak et al. [105]	Retrospective study	Comparison, through CBCT, of the results of floor lift with xenograft or use of PRF.	Thirty sinus elevations with simultaneous implantation were conducted using xenograft or PRF.	Promising results after 3 years of follow-up: PRF in sinus elevation offers effective alternatives for soft tissue management.
J Romero-Millán et al. [106]	Cohort study	Comparison of direct SL with simultaneous or delayed implantation or placement in native bone, after 5 years of follow-up.	Study on posterior maxillary implant treatment, comparing three approaches. Analysis of bone ridge, loss, and implant success after 5 years. 163 patients, 329 implants. Mean follow-up 7 years.	Similarity in bone and implant success. Higher resorption in first 12 months, stability at 5 years. Similar success between implants in native bone and with sinus lift. Graft reduction in first 12 months, stabilization at 5 years.
Bo Molemans et al. [107]	Cohort study	The study evaluates the use of L-PRF for grafting in sinus lift and SIP, demonstrating promising results.	The single-cohort study evaluated newly formed bone by CBCT after dental implant and abutment. Twenty-two transalveolar and six lateral sinus floor elevation were performed in 26 patients.	The use of L-PRF for simultaneous sinus floor elevations and implant offers convenience, safety, and cost-effectiveness, promoting natural bone formation.
Vasileios Ntontoulos et al. [108]	Clinical trial	Innovative protocol with CGF and piezosurgery to increase sinus floor offers a minimally invasive alternative with reduced morbidity.	Study of five patients shows efficacy of the piezo-albumin-concentrated growth factor (Alb-CGF) protocol to augment residual bone with minimal invasiveness.	Eight implants without complications, complete osseointegration, and bone formation after 4–6 months, with positive correlation between implant stability and protocol. The protocol with Alb-CGF promotes bone regeneration, reduces postoperative morbidity, and accelerates healing, with safe membrane lift and implant fixation.
Eduardo Anitua et al. [109]	Retrospective study	Analyzing height changes around short implants after transcrestal sinus lift with growth factor-rich plasma.	Study of 26 patients with atrophic jaws: placement of 41 short implants with transcrestal sinus augmentation.	Considerable bone augmentation over time, stability of apical height.
RS Pereira et al. [110]	Clinical trial	Comparison *β*-TCP with autologous grafts and their mixing in sinus lift, evaluating their surgical and regenerative efficacy.	Bone transplantation with *β*-TCP alone or with autologous bone.	Immunostaining reveals that *β*-TCP stimulates cellular activity and immature bone, suggesting potential as a bone substitute.
Iya H Ghassib et al. [111]	Case report	Solving a clinical case of a 38-year-old man with complications related to postdental extraction (root in sinus, fenestration, low RBH) by rehabilitating him with a dental implant.	Use of human platelet-derived growth factor-BB (rh-PDGF-BB) with dental implant for sinus lift, without graft, by partial flap technique.	At 8 months, there was a vertical bone gain of 6.2 mm and complete implant coverage.
Nghiem Doan et al. [112]	Case report	Two-case study using PBM, PES, and CGF in implant sinus lift to promote angiogenesis and tissue regeneration, promoting the effectiveness of the dental implant procedure.	Two-case study: combined use of PBM, PES, and CGF in implant sinus lift.	Accelerated angiogenesis, complication-free healing, and 100% implant success rate after 10 months were promoted.
Silvio Taschieri et al. [113]	Case report	Split-mouth study: comparison of platelet concentrates graft and bovine bone mineral versus bovine bone mineral alone in sinus lift.	Study of six edentulous patients with histologic analysis versus a test group.	Use of platelet-rich plasma with bovine bone mineral shows no significant difference in viable bone formation in sinus lift.
Cristian Adrian Raţiu et al. [114]	Case report	Case report of sinus membrane elevation performed with PRGF and fibrin clot, followed by bone regeneration after 8 months.	Sinus membrane lift and complications with vestibular access. Removal and subsequent bone regeneration with PRGF and fibrin clot.	Promising results of sinus lift with PRGF and fibrin clot require further clinical studies for well-founded scientific conclusions.

## Data Availability

The data sharing is not applicable to this article as no datasets were generated or analyzed during the current study.

## Nomenclature

Alb-CGF: Albumin-concentrated growth factor
BDGFs: Blood-derived growth factors
BMP: Bone morphogenetic protein
*β*-TCP: Beta-tricalcium phosphate
CBCT: Cone beam computed tomography
CGF: Concentrated growth factor
cHA: Carbonated hydroxyapatite
DBBM: Deproteinized bovine bone mineral
L-PRF: Leukocyte-rich fibrin
MSL: Maxillary sinus lift
OS: Oral surgery
OSFE: Osteotome sinus floor elevation
PBM: Photobiomodulation
PDGF: Platelet-derived growth factor
PES: Piezoelectric surgery
PRF: Platelet-rich fibrin
PRGFs: Plasma rich in growth factors
PRP: Platelet-rich plasma
RBH: Residual bone height
rhPDGF-BB: Human platelet-derived growth factor-BB
SL: Sinus lift
TSFE: Transalveolar sinus floor elevation.

## References

[1] Tavelli L., Barootchi S., Rodriguez M. V. (2022). Recombinant Human Platelet-Derived Growth Factor Improves Root Coverage of a Collagen Matrix for Multiple Adjacent Gingival Recessions: A Triple-Blinded, Randomized, Placebo-Controlled Trial. *Journal of Clinical Periodontology*.

[2] Tavelli L., Barootchi S., Rasperini G., Giannobile W. V. (2023). Clinical and Patient-Reported Outcomes of Tissue Engineering Strategies for Periodontal and Peri-Implant Reconstruction. *Periodontology 2000*.

[3] Gupta R., Gupta N., Weber D. D. S. (2024). Dental Implants. *StatPearls*.

[4] Inchingolo F., Tatullo M., Marrelli M. (2010). Trial with Platelet-Rich Fibrin and Bio-Oss Used as Grafting Materials in the Treatment of the Severe Maxillar Bone Atrophy: Clinical and Radiological Evaluations. *European Review for Medical and Pharmacological Sciences*.

[5] Swedish Council on Health Technology Assessment (2010). Prosthetic Rehabilitation of Partially Dentate or Edentulous Patients: A Systematic Review. http://www.ncbi.nlm.nih.gov/books/NBK448012/.

[6] Inchingolo F., Cantore S., Dipalma G. (2017). Platelet Rich Fibrin in the Management of Medication-Related Osteonecrosis of the Jaw: A Clinical and Histopathological Evaluation. *Journal of Biological Regulators and Homeostatic Agents*.

[7] Inchingolo A. M., Patano A., Pede C. D. (2023). Autologous Tooth Graft: Innovative Biomaterial for Bone Regeneration. Tooth Transformer and the Role of Microbiota in Regenerative Dentistry. A Systematic Review. *Journal of Functional Biomaterials*.

[8] Tatullo M., Marrelli M., Cassetta M. (2012). Platelet Rich Fibrin (P.R.F.) in Reconstructive Surgery of Atrophied Maxillary Bones: Clinical and Histological Evaluations. *International Journal of Medical Sciences*.

[9] Orjonikidze R., Orjonikidze Z., Shirokov I., Arutyunov S. (2018). Non-Removable Denture Prototypes, Effective in Dental Implantation. *Georgian Medical News*.

[10] Inchingolo F., Inchingolo A. M., Latini G. (2023). Application of Graphene Oxide in Oral Surgery: A Systematic Review. *Materials*.

[11] Balzanelli M. G., Distratis P., Dipalma G. (2021). Sars-CoV-2 Virus Infection May Interfere CD34+ Hematopoietic Stem Cells and Megakaryocyte–Erythroid Progenitors Differentiation Contributing to Platelet Defection Towards Insurgence of Thrombocytopenia and Thrombophilia. *Microorganisms*.

[12] Paul C., Bagshaw S., Bonita R. (1991). 1991 Cervical Screening Recommendations: A Working Group Report. *The New Zealand medical journal*.

[13] Tao B., Yu X., Wang W. (2023). A Deep Learning-Based Automatic Segmentation of Zygomatic Bones From Cone-Beam Computed Tomography Images: A Proof of Concept. *Journal of Dentistry*.

[14] Sabbah A., Romanos G., Delgado-Ruiz R. (2022). Extended Post-Curing Light Exposure and Sandblasting Effects on Surface Hydrophobicity of 3D-Printed Denture Base Resin. *Prosthesis*.

[15] Fiorillo L., Milone D., D’Andrea D. (2022). Finite Element Analysis of Zirconia Dental Implant. *Prosthesis*.

[16] Choi H., Hong M.-H. (2023). Implant Restoration Using a New Cementless Screw-Retained Type Prosthetic (TDP) System: Case Series. *Prosthesis*.

[17] Pasta S. (2023). In Silico Analysis of the MitraClip in a Realistic Human Left Heart Model. *Prosthesis*.

[18] Louessard A., Bonnet X., Catapano A., Pillet H. (2022). Quantification of the Influence of Prosthetic Ankle Stiffness on Static Balance Using Lower Limb Prosthetic Simulators. *Prosthesis*.

[19] Inchingolo F., Hazballa D., Inchingolo A. D. (2022). Innovative Concepts and Recent Breakthrough for Engineered Graft and Constructs for Bone Regeneration: A Literature Systematic Review. *Materials*.

[20] Favia G., Tempesta A., Limongelli L., Crincoli V., Piattelli A., Maiorano E. (2015). Metastatic Breast Cancer in Medication-Related Osteonecrosis Around Mandibular Implants. *American Journal of Case Reports*.

[21] Mittal Y., Jindal G., Garg S. (2016). Bone Manipulation Procedures in Dental Implants. *Indian Journal of Dentistry*.

[22] Demetriades N., Park J., Laskarides C. (2011). Alternative Bone Expansion Technique for Implant Placement in Atrophic Edentulous Maxilla and Mandible. *Journal of Oral Implantology*.

[23] Minervini G., Franco R., Marrapodi M. M., Fiorillo L., Cervino G., Cicciù M. (2023). The Association Between Parent Education Level, Oral Health, and Oral-Related Sleep Disturbance. An Observational Crosssectional Study. *European Journal of Paediatric Dentistry*.

[24] Minervini G., Marrapodi M. M., Cicciù M. (2023). Online Bruxism-Related Information: Can People Understand What They Read? A Cross-Sectional Study. *Journal of Oral Rehabilitation*.

[25] Minervini G., Franco R., Marrapodi M. M., Fiorillo L., Cervino G., Cicciù M. (2023). Post-Traumatic Stress, Prevalence of Temporomandibular Disorders in War Veterans: Systematic Review With Meta-Analysis. *Journal of Oral Rehabilitation*.

[26] Almeida L. E., Cicciù M., Doetzer A., Beck M. L., Cervino G., Minervini G. (2023). Mandibular Condylar Hyperplasia and Its Correlation With Vascular Endothelial Growth Factor. *Journal of Oral Rehabilitation*.

[27] Minervini G., Franco R., Marrapodi M. M., Almeida L. E., Ronsivalle V., Cicciù M. (2023). Prevalence of Temporomandibular Disorders (TMD) in Obesity Patients: A Systematic Review and Meta-Analysis. *Journal of Oral Rehabilitation*.

[28] Uzunçıbuk H., Marrapodi M. M., Meto A., Ronsivalle V., Cicciù M., Minervini G. (2024). Prevalence of Temporomandibular Disorders in Clear Aligner Patients Using Orthodontic Intermaxillary Elastics Assessed With Diagnostic Criteria for Temporomandibular Disorders (DC/TMD) Axis II Evaluation: A Cross-Sectional Study. *Journal of Oral Rehabilitation*.

[29] Minervini G., Franco R., Marrapodi M. M., Di Blasio M., Isola G., Cicciù M. (2023). Conservative Treatment of Temporomandibular Joint Condylar Fractures: A Systematic Review Conducted According to PRISMA Guidelines and the Cochrane Handbook for Systematic Reviews of Interventions. *Journal of Oral Rehabilitation*.

[30] Minervini G., Franco R., Marrapodi M. M., Di Blasio M., Ronsivalle V., Cicciù M. (2023). Children Oral Health and Parents Education Status: A Cross Sectional Study. *BMC Oral Health*.

[31] Scandurra C., Gasparro R., Dolce P. (2021). The Role of Cognitive and Non-Cognitive Factors in Dental Anxiety: A Mediation Model. *European Journal of Oral Sciences*.

[32] D’Esposito V., Lecce M., Marenzi G. (2020). Platelet-Rich Plasma Counteracts Detrimental Effect of High-Glucose Concentrations on Mesenchymal Stem Cells From Bichat Fat Pad. *Journal of Tissue Engineering and Regenerative Medicine*.

[33] Del Amo F. S. L., Yu S.-H., Sammartino G. (2020). Peri-Implant Soft Tissue Management: Cairo Opinion Consensus Conference. *International Journal of Environmental Research and Public Health*.

[34] Sammartino G., Marenzi G., Howard C. M. (2008). Chondrosarcoma of the Jaw: A Closer Look at its Management. *Journal of Oral and Maxillofacial Surgery*.

[35] Piombino P., Marenzi G., Dell’Aversana Orabona G., Califano L., Sammartino G. (2015). Autologous Fat Grafting in Facial Volumetric Restoration. *Journal of Craniofacial Surgery*.

[36] Ahmed N., Humayun M., Abbasi M., Jamayet N., Habib S., Zafar M. (2021). Comparison of Canine-Guided Occlusion With Other Occlusal Schemes in Removable Complete Dentures: A Systematic Review. *Prosthesis*.

[37] Bori E., Deslypere C., Estaire Muñoz L., Innocenti B. (2023). Clinical Results of the Use of Low-Cost TKA *Prosthesis* in Low Budget Countries—A Narrative Review. *Prosthesis*.

[38] Marrapodi M. M., Mascolo A., di Mauro G., Mondillo G., Pota E., Rossi F. (2022). The Safety of Blinatumomab in Pediatric Patients With Acute Lymphoblastic Leukemia: A Systematic Review and Meta-Analysis. *Frontiers in Pediatrics*.

[39] Di Paola A., Tortora C., Argenziano M., Marrapodi M. M., Rossi F. (2022). Emerging Roles of the Iron Chelators in Inflammation. *International Journal of Molecular Sciences*.

[40] Arrigoni R., Ballini A., Santacroce L. (2022). Another Look at Dietary Polyphenols: Challenges in Cancer Preventionand Treatment. *Current Medicinal Chemistry*.

[41] Santacroce L., Di Cosola M., Bottalico L. (2021). Focus on HPV Infection and the Molecular Mechanisms of Oral Carcinogenesis. *Viruses*.

[42] Rapone B., Inchingolo A. D., Trasarti S. (2022). Long-Term Outcomes of Implants Placed in Maxillary Sinus Floor Augmentation With Porous Fluorohydroxyapatite (Algipore FRIOS) in Comparison With Anorganic Bovine Bone (Bio-Oss) and Platelet Rich Plasma (PRP): A Retrospective Study. *Journal of Clinical Medicine*.

[43] Inchingolo A. D., Inchingolo A. M., Bordea I. R. (2021). The Effectiveness of Osseodensification Drilling Protocol for Implant Site Osteotomy: A Systematic Review of the Literature and Meta-Analysis. *Materials*.

[44] Stacchi C., Spinato S., Lombardi T. (2020). Minimally Invasive Management of Implant-Supported Rehabilitation in the Posterior Maxilla, Part II. *The International Journal of Periodontics & Restorative Dentistry*.

[45] Anand M., Panwar S. (2021). Role of Navigation in Oral and Maxillofacial Surgery: A Surgeon’s Perspectives. *Clinical, Cosmetic and Investigational Dentistry*.

[46] Inchingolo A. D., Pezzolla C., Patano A. (2022). Experimental Analysis of the Use of Cranial Electromyography in Athletes and Clinical Implications. *International Journal of Environmental Research and Public Health*.

[47] Han S.-J. (2023). The Role of Oral and Maxillofacial Surgeons in Maxillary Sinus Diseases Related to Dental Implants. *Journal of the Korean Association of Oral and Maxillofacial Surgeons*.

[48] Kang S.-H., Kim M.-K., Kim J.-H., Park H.-K., Park W. (2012). Marker-Free Registration for the Accurate Integration of CT Images and the Subject’s Anatomy During Navigation Surgery of the Maxillary Sinus. *Dentomaxillofacial Radiology*.

[49] Bromberg N., Brizuela M. (2024). Dental Cone Beam Computed Tomography. *StatPearls*.

[50] Inchingolo A. D., Patano A., Coloccia G. (2021). Genetic Pattern, Orthodontic and Surgical Management of Multiple Supplementary Impacted Teeth in a Rare, Cleidocranial Dysplasia Patient: A Case Report. *Medicina*.

[51] Morgan N., Meeus J., Shujaat S., Cortellini S., Bornstein M. M., Jacobs R. (2023). CBCT for Diagnostics, Treatment Planning and Monitoring of Sinus Floor Elevation Procedures. *Diagnostics*.

[52] Blasi A., Nucera R., Ronsivalle V., Candida E., Grippaudo C. (2022). Asymmetry Index for the Photogrammetric Assessment of Facial Asymmetry. *American Journal of Orthodontics and Dentofacial Orthopedics*.

[53] Lo Russo L., Guida L., Mariani P. (2023). Effect of Fabrication Technology on the Accuracy of Surgical Guides for Dental-Implant Surgery. *Bioengineering*.

[54] Gasparro R., Adamo D., Masucci M., Sammartino G., Mignogna M. D. (2019). Use of Injectable Platelet-Rich Fibrin in the Treatment of Plasma Cell Mucositis of the Oral Cavity Refractory to Corticosteroid Therapy: A Case Report. *Dermatologic Therapy*.

[55] Caggiano M., Gasparro R., D’Ambrosio F., Pisano M., Di Palo M. P., Contaldo M. (2022). Smoking Cessation on Periodontal and Peri-Implant Health Status: A Systematic Review. *Dentistry Journal*.

[56] Kao S.-Y., Lui M.-T., Cheng D.-H., Chen T.-W. (2015). Lateral Trap-Door Window Approach With Maxillary Sinus Membrane Lifting for Dental Implant Placement in Atrophied Edentulous Alveolar Ridge. *Journal of the Chinese Medical Association*.

[57] 10 SINUS LIFT PROCEDURES seminar 10.pptx (2023). SlideShare. September 28. https://www.slideshare.net/slideshow/10-sinus-lift-procedures-seminar-10pptx/261540670.

[58] Laudadio C., Inchingolo A. D., Malcangi G. (2021). Management of Anterior Open-Bite in the Deciduous, Mixed and Permanent Dentition Stage: A Descriptive Review. *Journal of Biological Regulators and Homeostatic Agents*.

[59] Aly L. (2018). Piezoelectric Surgery: Applications in Oral & Maxillofacial Surgery. *Future Dental Journal*.

[60] Cheon K.-J., Yang B.-E., Cho S.-W., Chung S.-M., Byun S.-H. (2020). Lateral Window Design for Maxillary Sinus Graft Based on the Implant Position. *International Journal of Environmental Research and Public Health*.

[61] Palermo A., Giannotti L., Di Chiara Stanca B. (2022). Use of CGF in Oral and Implant Surgery: From Laboratory Evidence to Clinical Evaluation. *International Journal of Molecular Sciences*.

[62] Laforgia A., Corsalini M., Stefanachi G., Pettini F., Di Venere D. (2015). Assessment of Psychopatologic Traits in a Group of Patients With Adult Chronic Periodontitis: Study on 108 Cases and Analysis of Compliance During and After Periodontal Treatment. *International Journal of Medical Sciences*.

[63] Corsalini M., Di Venere D., Stefanachi G. (2017). Maxillary Overdenture Retained With an Implant Support CAD-CAM Bar: A 4 Years Follow Up Case. *The Open Dentistry Journal*.

[64] Laforgia A., Corsalini M., Stefanachi G. (2016). Non-Surgical Periodontal Management in Scleroderma Disease Patients. *Journal of Biological Regulators and Homeostatic Agents*.

[65] DENTAL SUPPLEMENT, Minetti E., Palermo A. (2019). Autologous Tooth Graft: A Histological Comparison Between Dentin Mixed With Xenograft and Dentin Alone Grafts in Socket Preservation. *Journal of Biological Regulators and Homeostatic Agents*.

[66] Franco R., Bollero P. (2021). Melatonin as an Index of Periodontal Disease. *European Journal of General Dentistry*.

[67] Bollero P., Arcuri L., Miranda M., Ottria L., Franco R., Barlattani A. (2017). Marfan Syndrome: Oral Implication and Management. *Oral & Implantology*.

[68] Franco R., Lupi E., Iacomino E. (2023). Low-Level Laser Therapy for the Treatment of Oral Mucositis Induced by Hematopoietic Stem Cell Transplantation: A Systematic Review With Meta-Analysis. *Medicina (Kaunas)*.

[69] Lumbau A. I., Meloni S. M., Tallarico M. (2021). Implant Placement Following Crestal Sinus Lift With Sequential Drills and Osteotomes: Five Years After Final Loading Results From a Retrospective Study. *Journal of Functional Biomaterials*.

[70] Bathla S. C., Fry R. R., Majumdar K. (2018). Maxillary Sinus Augmentation. *Journal of Indian Society of Periodontology*.

[71] Ferraz M. P. (2023). Bone Grafts in Dental Medicine: An Overview of Autografts, Allografts and Synthetic Materials. *Materials*.

[72] Ferrazzano G. F., Sangianantoni S., Mitrano R. L., Ingenito A., Alcidi B., Cantile T. (2019). Assessing Changes in Oral Health-Related Quality of Life and Body Growth in 3–5 Years Old Children Following Dental Treatment Under General Anaesthesia due to Severe Dental Caries. *European Journal of Paediatric Dentistry*.

[73] Stacchi C., Spinato S., Lombardi T. (2020). Minimally Invasive Management of Implant-Supported Rehabilitation in the Posterior Maxilla, Part I. Sinus Floor Elevation: Biologic Principles and Materials. *The International Journal of Periodontics & Restorative Dentistry*.

[74] Cha H.-S., Kim J.-W., Hwang J.-H., Ahn K.-M. (2016). Frequency of Bone Graft in Implant Surgery. *Maxillofacial Plastic and Reconstructive Surgery*.

[75] Chatelet M., Afota F., Savoldelli C. (2022). Review of Bone Graft and Implant Survival Rate: A Comparison Between Autogenous Bone Block Versus Guided Bone Regeneration. *Journal of Stomatology, Oral and Maxillofacial Surgery*.

[76] Bone Augmentation Procedures in Implantology | SpringerLink. https://link.springer.com/chapter/10.1007/978-981-15-1346-6_19.

[77] Stacchi C., Rapani A., Lombardi T., Bernardello F., Nicolin V., Berton F. (2022). Does New Bone Formation Vary in Different Sites Within the Same Maxillary Sinus After Lateral Augmentation? A Prospective Histomorphometric Study. *Clinical Oral Implants Research*.

[78] Rosa A., Ranieri N., Miranda M., Mehta V., Fiorillo L., Cervino G. (2024). Mini Crestal Sinus Lift With Bone Grafting and Simultaneous Insertion of Implants in Severe Maxillary Conditions as an Alternative to Lateral Sinus Lift: Multicase Study Report of Different Techniques. *Journal of Craniofacial Surgery*.

[79] Comuzzi L., Tumedei M., Petrini M. (2023). Clinical and Radiological Evaluation of a Self-Condensing Bone Implant in One-Stage Sinus Augmentation: A 3-Year Follow-Up Retrospective Study. *International Journal of Environmental Research and Public Health*.

[80] Chiapasco M., Abati S., Romeo E., Vogel G. (1999). Clinical Outcome of Autogenous Bone Blocks or Guided Bone Regeneration With e-PTFE Membranes for the Reconstruction of Narrow Edentulous Ridges. *Clinical Oral Implants Research*.

[81] Fang D., Long Z., Hou J. (2020). Clinical Application of Concentrated Growth Factor Fibrin Combined With Bone Repair Materials in Jaw Defects. *Journal of Oral and Maxillofacial Surgery*.

[82] Pasini M., Giuca M. R., Ligori S. (2020). Association Between Anatomical Variations and Maxillary Canine Impaction: A Retrospective Study in Orthodontics. *Applied Sciences*.

[83] Scorzetti L., Marcattili D., Pasini M., Mattei A., Marchetti E., Marzo G. (2013). Association Between Obesity and Periodontal Disease in Children. *European Journal of Paediatric Dentistry*.

[84] Quinzi V., Mummolo S., Bertolazzi F., Campanella V., Marzo G., Marchetti E. (2020). Comparison of Mandibular Arch Expansion by the Schwartz Appliance Using Two Activation Protocols: A Preliminary Retrospective Clinical Study. *Journal of Functional Morphology and Kinesiology*.

[85] Saccomanno S., Quinzi V., D’Andrea N., Albani A., Coceani Paskay L., Marzo G. (2021). Traumatic Events and Eagle Syndrome: Is There Any Correlation? A Systematic Review. *Healthcare*.

[86] Śmielecka M., Dorocka-Bobkowska B. (2022). Comparison of Two Optical Devices Used for Artificial Tooth Color Selection. *Dental and Medical Problems*.

[87] Sakai T., Li H., Shimada T. (2023). Development of Artificial Intelligence Model for Supporting Implant Drilling Protocol Decision Making. *Journal of Prosthodontic Research*.

[88] Maniaci A., La Mantia I., Mayo-Yáñez M. (2023). Vocal Rehabilitation and Quality of Life After Total Laryngectomy: State-of-the-Art and Systematic Review. *Prosthesis*.

[89] D’Addazio G., Xhajanka E., Cerone P. (2021). Traditional Removable Partial Dentures Versus Implant-Supported Removable Partial Dentures: A Retrospective, Observational Oral Health-Related Quality-of-Life Study. *Prosthesis*.

[90] Al-Aroomi O. A., Ou Y., Sakran K. A. (2024). Effectiveness of Concentrated Growth Factors With or Without Grafting Materials in Maxillary Sinus Augmentation: A Systematic Review. *BMC Oral Health*.

[91] Qiu P., Zhang X., Cao R., Xu H., Jiang Z., Lei J. (2024). Assessment of the Efficacy of Autologous Blood Preparations in Maxillary Sinus Floor Elevation Surgery: A Systematic Review and Meta-Analysis. *BMC Oral Health*.

[92] Bacevich B. M., Smith R. D. J., Reihl A. M., Mazzocca A. D., Hutchinson I. D. (2024). Advances With Platelet-Rich Plasma for Bone Healing. *Biologics: Targets and Therapy*.

[93] Malcangi G., Patano A., Palmieri G. (2023). Maxillary Sinus Augmentation Using Autologous Platelet Concentrates (Platelet-Rich Plasma, Platelet-Rich Fibrin, and Concentrated Growth Factor) Combined With Bone Graft: A Systematic Review. *Cells*.

[94] Badran Z., Abdallah M.-N., Torres J., Tamimi F. (2018). Platelet Concentrates for Bone Regeneration: Current Evidence and Future Challenges. *Platelets*.

[95] Ferrazzano G. F., Sangianantoni G., Cantile T., Amato I., Ingenito A., Noschese P. (2012). Dental Health in Asthmatic Children: A South Italy Study. *Journal of Dentistry for Children (Chicago, Illinois)*.

[96] Page M. J., McKenzie J. E., Bossuyt P. M. (2021). The PRISMA 2020 Statement: An Updated Guideline for Reporting Systematic Reviews. *The British Medical Journal*.

[97] Younes F., Cosyn J., De Bruyckere T., Cleymaet R., Eghbali A. (2019). A 2-Year Prospective Case Series on Volumetric Changes, PROMs, and Clinical Outcomes Following Sinus Floor Elevation Using Deproteinized Bovine Bone Mineral as Filling Material. *Clinical Implant Dentistry and Related Research*.

[98] Adalı E., Yüce M. O., Günbay T., Günbay S. (2021). Does Concentrated Growth Factor Used With Allografts in Maxillary Sinus Lifting Have Adjunctive Benefits?. *Journal of Oral and Maxillofacial Surgery*.

[99] Gurler G., Delilbasi C. (2016). Effects of Leukocyte-Platelet Rich Fibrin (L-PRF) on Post-Operative Complications of Direct Sinus Lifting. *Minerva Stomatol*.

[100] Sher Ling S. L. (2018). CRNC13: Healing Large Bone Defect With Concentrated Growth Factor in Window Sinus Lift: A Case Report. *The Journal of Indian Prosthodontic Society*.

[101] de Almeida Barros Mourão C. F., Lourenço E. S., Nascimento J. R. B. (2019). Does the Association of Blood-Derived Growth Factors to Nanostructured Carbonated Hydroxyapatite Contributes to the Maxillary Sinus Floor Elevation? A Randomized Clinical Trial. *Clinical Oral Investigations*.

[102] Ding Y., Wang X. (2018). Long-Term Effects of Bone Morphogenetic Protein-2-Loaded Calcium Phosphate on Maxillary Sinus Lift Surgery for Delayed and Simultaneous Dental Implantation. *Journal of Craniofacial Surgery*.

[103] Khouly I., Pardiñas López S., Aliaga I., Froum S. J. (2017). Long-Term Implant Survival After 100 Maxillary Sinus Augmentations Using Plasma Rich in Growth Factors. *Implant Dentistry*.

[104] Chen H., Zhou L., Wu D., Zhang J., Zheng Y., Chen Y. (2022). Osteotome Sinus Floor Elevation With Concentrated Growth Factor and Simultaneous Implant Placement With or Without Bone Grafting: A Retrospective Study. *International Journal of Oral and Maxillofacial Surgery*.

[105] Dominiak S., Karuga-Kuźniewska E., Popecki P., Kubasiewicz-Ross P. (2021). PRF versus Xenograft in Sinus Augmentation in Case of HA-Coating Implant Placement: A 36-Month Retrospective Study. *Advances in Clinical and Experimental Medicine*.

[106] Romero-Millán J., Hernández-Alfaro F., Peñarrocha-Diago M., Soto-Peñaloza D., Peñarrocha-Oltra D., Peñarrocha-Diago M. A. (2018). Simultaneous and Delayed Direct Sinus Lift Versus Conventional Implants: Retrospective Study With 5-Years Minimum Follow-up. *Medicina Oral, Patología Oral y Cirugía Bucal*.

[107] Molemans B., Cortellini S., Jacobs R., Teughels W., Pinto N., Quirynen M. (2019). Simultaneous Sinus Floor Elevation and Implant Placement Using Leukocyte- and Platelet-Rich Fibrin as a Sole Graft Material. *The International Journal of Oral & Maxillofacial Implants*.

[108] Ntontoulos V., Dabarakis N. (2024). The Effect of Denatured Albumin With Concentrated Growth Factors in Minimally Invasive Sinus Piezosurgery: Preliminary Pilot Study Results. *European Journal of Dentistry*.

[109] Anitua E., Flores J., Alkhraisat M. H. (2016). Transcrestal Sinus Lift Using Platelet Concentrates in Association to Short Implant Placement: A Retrospective Study of Augmented Bone Height Remodeling. *Clinical Implant Dentistry and Related Research*.

[110] Pereira R. S., Gorla L. F., Boos F. B. J. D., Okamoto R., Garcia Júnior I. R., Hochuli-Vieira E. (2017). Use of Autogenous Bone and Beta-Tricalcium Phosphate in Maxillary Sinus Lifting: Histomorphometric Study and Immunohistochemical Assessment of RUNX2 and VEGF. *International Journal of Oral and Maxillofacial Surgery*.

[111] Ghassib I. H., Saleh M. H. A., Wang H. L. (2024). Human Platelet-Derived Growth Factor-BB (rhPDGF-BB) With Collagen Matrix for Sinus Elevation Without Using a Bone Graft. *Clinical Advances in Periodontics*.

[112] Doan N., Huynh T. Q., Tran S. (2020). Multidisciplinary Approach to Maximize Angiogenesis and Wound Healing Using Piezoelectric Surgery, Concentrated Growth Factors and Photobiomodulation for Dental Implant Placement Surgery Involving Lateral Wall Sinus Lift: Two Case Reports. *Vascular Cell*.

[113] Taschieri S., Testori T., Corbella S. (2015). Platelet-Rich Plasma and Deproteinized Bovine Bone Matrix in Maxillary Sinus Lift Surgery: A Split-Mouth Histomorphometric Evaluation. *Implant Dentistry*.

[114] Raţiu C. A., Zdrîncă M. M., Boşca A. B., Ruxanda F., Miclăuş V., Ilea A. (2018). The Effect of Plasma Rich in Growth Factors in Bone Augmentation After Sinus Lift Complications: A Case Report. *Romanian Journal of Morphology and Embryology*.

[115] Alshamrani A. M., Mubarki M., Alsager A. S., Alsharif H. K., AlHumaidan S. A., Al-Omar A. (2023). Maxillary Sinus Lift Procedures: An Overview of Current Techniques, Presurgical Evaluation, and Complications. *Cureus*.

[116] Kühl S., Payer M., Kirmeier R., Wildburger A., Wegscheider W., Jakse N. (2014). The Influence of Bone Marrow Aspirates and Concentrates on the Early Volume Stability of Maxillary Sinus Grafts With Deproteinized Bovine Bone Mineral—First Results of a RCT. *Clinical Oral Implants Research*.

[117] Wu X., Ye M., Sun J., Yan Q., Shi B., Xia H. (2022). Patient-Reported Outcome Measures Following Surgeries in Implant Dentistry and Associated Factors: A Cross-Sectional Study. *BMJ Open*.

[118] McGrath C., Lam O., Lang N. (2012). An Evidence-Based Review of Patient-Reported Outcome Measures in Dental Implant Research Among Dentate Subjects. *Journal of Clinical Periodontology*.

[119] Ghasemirad M., Chitsazi M. T., Faramarzi M., Roshangar L., Babaloo A., Chitsazha R. (2023). Histological Examination of the Effect of Concentrated Growth Factor (CGF) on Healing Outcomes After Maxillary Sinus Floor Augmentation Surgery. *Journal of Medicine and Life*.

[120] Españo E., Kim J., Kim J.-K. (2022). Utilization of Aloe Compounds in Combatting Viral Diseases. *Pharmaceuticals*.

[121] Chen L., Cheng J., Cai Y., Zhang J., Yin X., Luan Q. (2023). Efficacy of Concentrated Growth Factor (CGF) in the Surgical Treatment of Oral Diseases: A Systematic Review and Meta-Analysis. *BMC Oral Health*.

[122] Chen C.-M., Hsu H.-J., Hsu K.-J., Tseng Y.-C. (2022). Clinical Significance of Postoperative Skeletal Relapse in the Treatment of Mandibular Prognathism: Receiver Operating Characteristic Curve Analysis. *Journal of the Formosan Medical Association*.

[123] Simonpieri A., Choukroun J., Corso M. D., Sammartino G., Ehrenfest D. M. D. (2011). Simultaneous Sinus-Lift and Implantation Using Microthreaded Implants and Leukocyte- and Platelet-Rich Fibrin as Sole Grafting Material: A 6-Year Experience. *Implant Dentistry*.

[124] Diss A., Dohan D., Mouhyi J., Mahler P. (2008). Osteotome Sinus Floor Elevation Using Choukroun’s Platelet-Rich Fibrin as Grafting Material: A 1-Year Prospective Pilot Study With Microthreaded Implants. *Oral Surgery, Oral Medicine, Oral Pathology, Oral Radiology, and Endodontology*.

[125] Gruber R. M., Ludwig A., Merten H.-A., Pippig S., Kramer F.-J., Schliephake H. (2009). Sinus Floor Augmentation With Recombinant Human Growth and Differentiation Factor-5 (rhGDF-5): A Pilot Study in the Goettingen Miniature Pig Comparing Autogenous Bone and rhGDF-5. *Clinical Oral Implants Research*.

[126] Gasparro R., Di Lauro A. E., Campana M. D. (2024). Effectiveness of Autologous Platelet Concentrates in the Sinus Lift Surgery: Findings From Systematic Reviews and Meta-Analyses. *Dentistry Journal*.

[127] Barakat S. O., Tawfik O. K., Kholy S. E., ElNahass H. (2024). Evaluation of Advanced Platelet-Rich Fibrin Compared to Subepithelial Connective Tissue Graft in the Surgical Management of Interdental Papilla Recession: A Randomized Controlled Trial. *Clinical Oral Investigations*.

